# A range division and contraction approach for nonconvex quadratic program with quadratic constraints

**DOI:** 10.1186/s40064-016-2735-y

**Published:** 2016-07-12

**Authors:** Chunshan Xue, Hongwei Jiao, Jingben Yin, Yongqiang Chen

**Affiliations:** School of Mathematics and Statistics, Zhoukou Normal University, Zhoukou, 466001 China; School of Mathematical Sciences, Henan Institute of Science and Technology, Xinxiang, 453003 China; College of Mathematics and Information Science, Henan Normal University, Xinxiang, 453007 China

**Keywords:** Nonconvex quadratic program with quadratic constraints, Global optimization, Underestimating linear relaxation, Range contraction approach, Branch and bound scheme, 90C20, 90C26, 65K05

## Abstract

This paper presents a novel range division and contraction approach for globally solving nonconvex quadratic program with quadratic constraints. By constructing new underestimating linear relaxation functions, we can transform the initial nonconvex quadratic program problem into a linear program relaxation problem. By employing a branch and bound scheme with a range contraction approach, we describe a novel global optimization algorithm for effectively solving nonconvex quadratic program with quadratic constraints. Finally, the global convergence of the proposed algorithm is proved and numerical experimental results demonstrate the effectiveness of the proposed approach.

## Background

The mathematical modeling of nonconvex quadratic program with quadratic constraints can be formulated as follows:

$$\begin{aligned} {\mathrm{(NQPQC)}}:\left\{ \begin{array}{ll} {\mathrm{min}} &{} F_{0}(x)=x^{T}Q_{0}x+d_{0}^{T}x\\ {\mathrm{s.t. }}&{} F_{i}(x)=x^{T}Q_{i}x+d_{i}^{T}x\le b_{i},\quad i=1,\ldots ,m,\\ &{}x\in X^{0}=\{x\in R^n:l^{0}\le x\le u^{0}\}, \end{array} \right. \end{aligned}$$

where $Q_{i}, i=0,1,\ldots ,m,$ are all symmetric $n\times n$ matrices, $d_0, d_{i}\in R^{n}, b_{i}\in R, i=1,\ldots ,m; l^{0}=(l^{0}_1,\ldots ,l^{0}_n)^T, u^{0}=(u^{0}_1,\ldots ,u^{0}_n)^T.$

Nonconvex quadratic program with quadratic constraints is worthy of study. On the one hand, this is because the kind of problems have many applications in practical problems, such as, heat exchanger engineering design, financial optimization, image processing, management science, etc (see Floudas and Visweswaran 1995; Shen 2007; Horst and Tuy 1996; Jiao and Liu in press; Bajirov and Rubinov 2001; Konno and Wijayanayake 2001; Sherali and Smith 1997). On the other hand, it is since a large number of nonlinear programming problems can be converted into this mathematical modeling, and the solutions of many nonlinear optimization problems can be approximated or obtained by solving a sequence of nonconvex quadratic programs with quadratic constraints. Moreover, from research point of view, since the nonconvex quadratic programs with quadratic constraints possess multiple local minimum points which are not globally minimum point, they exist significant computational and theoretical challenges. Therefore, it is necessary to put forward a new global optimization approach for solving the nonconvex quadratic program with quadratic constraints.

In past several decades, various algorithms have been developed for solving the nonconvex quadratic program with quadratic constraints and its special form, which are given as follows. Based on Newton method, branching rule and cutting plane, Vandenbussche and Nemhauser presented a branch-and-cut method for nonconvex quadratic programs with box constraints (Vandenbussche and Nemhauser 2005). Used different d.c. decomposition method to construct the relaxation of quadratic objective function, and used the “optimal level solution” parametrical approach to solve relaxation problem, Cambini and Sodini (2005) proposed a decomposition method for solving nonconvex quadratic programs over a compact polyhedral feasible region. By decomposing a large-scale quadratic programming into a serial of small-scale ones and approximating the solution of the large-scale quadratic programming via the solutions of these small-scale ones, Li and Zhang (2006) presented a decomposition algorithm for solving large-scale quadratic programming problems. Through decomposing quadratic objective function into a separable equivalent function, then by constructing linear under-estimator of the corresponding objective function, Shen et al. (2008) presented a decomposition and linearization method for globally solving quadratic programs with linear constraints. Vavasis (1992) presented an approximation algorithm for indefinite quadratic programming, and concluded that such an approximation solution can be found in polynomial time. Based on D.C. decomposition, Cholesky factorization and convex relaxation, Yajima and Fujie (1998) proposed a decomposition-and-relaxation algorithm for the general quadratic problems with box constraints. Ye (1992) proposed an affine-scaling algorithm to solve nonconvex indefinite or negative definite quadratic programs problems. By utilizing cutting plane technique, Gao and Deng (2008) presented a branch and bound method mixed with cutting plane technique for solving concave quadratic programming problems. Using lagrangian underestimates and interval Newton method, Voorhis (2002) proposed a global optimization algorithm for quadratic programs. Based on simplicial branch-and-bound scheme, Raber (1998) presented a simplicial branch-and-bound method for solving nonconvex quadratic programs. Based on parametric linear relaxation and new linearizing technique, Jiao et al. (2015), Jiao and Chen (2013) proposed two branch and bound algorithms for globally solving nonconvex quadratic programs. Using duality bounds and branch-and-bound scheme, Thoai (2000) presented a duality bound method for the general quadratic programming problem with quadratic constraints. Based on linear relaxation method and branch-and-bound framework with rectangle reducing technique, Shen and Liu (2008), Gao et al. (2005a, b), Jiao et al. (2014) proposed four branch-and-reduce algorithms for solving nonconvex quadratic programs, respectively. Based on branch-and-bound scheme, An and Tao (1997), An and Tao (1998) presented two D.C. algorithms for solving nonconvex quadratic programs. Apart from the above reviewed quadratic programs algorithms, many algorithms (see Shen and Jiao 2006; Wang and Liang 2005; Wang et al. 2004; Shen 2005; Shen and Li 2013; Shen and Bai 2013; Shen et al. 2009) for solving geometric programming can be also used to solve the nonconvex quadratic program with quadratic constraints proposed in this paper. In addition, some recent artificial intelligent optimization algorithms (Zhang et al. 2013, 2014a, b, 2016) are developed, which are also used to obtain local optimal solution of the nonconvex quadratic program with quadratic constraints.

The above most deterministic algorithms for solving nonconvex quadratic programs are based on relaxation technique and branch-and-bound scheme, since the exhaustiveness of branching rule leads to a significant increase in the computational burden for solving nonconvex quadratic programs, until today, there is short of more effective algorithm for solving such problems, so it is necessary to establish a good algorithm for the NQPQC. Therefore, the main motivation for the paper is to construct a novel linearized technique and a range contraction approach, and based on these techniques develop an effective algorithm for solving the NQPQC.

In this paper, a novel range division and contraction approach will be proposed for globally solving the nonconvex quadratic program with quadratic constraints. The main features of the proposed algorithm in this paper are as follows. Firstly, a novel linearized technique is constructed for systematically converting the NQPQC into a sequence of linear program problems, and the solutions of the converted linear program problems are used to approximate the solution of the NQPQC, which can be computed by using simplex approach. Secondly, in order to accelerate the running speed of our approach, a range contraction method is constructed and employed in our algorithm. Next, use branch-and-bound framework with a range contraction approach, a new global optimization algorithm is established. Finally, the global convergence of the proposed approach is proved and numerical experimental results demonstrate the computational efficiency of our algorithm.

The remaining contents of the paper are stated as follows. Second section describes a new linearized method and the linear program relaxation problem of the initial NQPQC is derived. In third section, a new range division and contraction approach is established and its global convergence is proved. Fourth section describes numerical results for some examples, which be computed by using the proposed algorithm. Finally some conclusions are drawn.

## New linearized approach

In this section, a new linearized approach is constructed for establishing the linear program relaxation problem of the NQPQC. Without loss of generality, let $X^k=\{x\in R^n|\ l^k=(l_1^k,\ldots ,l_n^k)^T\le x\le u^k=(u_1^k,\ldots ,u_n^k)^T\}\subseteq X^{0}$, and let $\lambda _i$ be the minimum eigenvalue of $Q_{i}$. Set

$$\begin{aligned} \theta _{i}=\left\{ \begin{array}{ll} 0,&{} \quad \text{ if } \ \lambda _i\ge 0, \\ |\lambda _i|+\rho ,&{} \quad \text{ if } \ \lambda _i< 0,\, \text{ where }\ \rho \ \text{ is } \text{ an } \text{ arbitrary } \text{ positive } \text{ real } \text{ number}. \end{array} \right. \end{aligned}$$

In the following, for any $i\in \{0,1,\ldots ,m\},$ for any $x\in X^k$, define

$$\begin{aligned} F^{L}_i(x)= & {} \left[ d_i+Q_i^{T}\left( l^{k}+u^{k}\right) +\theta _i\left( u^{k}-l^{k} \right) \right] ^Tx+2\theta _i\left( u^k\right) ^{T}l^k-\theta _i\left( u^k\right) ^{T}\left( u^k\right) \\&\quad -\left( \frac{l^{k}+u^k}{2}\right) ^T\left( Q_i+\theta _iI\right) \left( \frac{l^{k}+u^k}{2}\right) . \end{aligned}$$

### **Theorem 1**

*For any*$x\in X^k,$*consider the functions*$F_{i}(x)$*and*$F^{L}_i(x)$, *we have:*

(i) $$F^{L}_i(x)\le F_{i}(x),i=0,1,\ldots ,m;$$
(ii) *For each*$$i=0,1,\ldots ,m,$$$$\Vert F_{i}(x)-F^{L}_i(x)\Vert \rightarrow 0$$*as*$$\Vert u^{k}-l^{k}\Vert \rightarrow 0.$$

### *Proof*

(1) Consider the function $x_j^2$ over $[l_j^k,u_j^k]$, according to the mean value theorem, we can get

$$\begin{aligned} x_j^2=2\xi ^k_{j}\left( x_{j}-u^k_{j}\right) +\left( u^k_{j}\right) ^{2}\le 2l^k_{j}\left( x_{j}-u^k_{j}\right) +\left( u^k_{j}\right) ^{2} =2l^k_{j}x_{j}-2u^k_{j}l^k_{j}+\left( u^k_{j}\right) ^{2}, \end{aligned}$$

where $$\xi ^k_{j}=\alpha l^k_{j}+(1-\alpha )u^k_{j}, \alpha \in [0,1], j=1,\ldots ,n.$$

By the expressions of the functions $F_{i}(x)$ and $F^{L}_i(x)$, we can get that

$$\begin{aligned} F_{i}(x)&=   x^TQ_ix+d_i^Tx\\&=   \left( x-\frac{l^{k}+u^k}{2}\right) ^T\left( Q_i+\theta _iI\right) \left( x-\frac{l^{k}+u^k}{2}\right) \\&\quad +d_i^Tx-\theta _i\sum \limits _{j=1}^n x_j^2+\left( l^{k}+u^k\right) ^T\left( Q_i+\theta _iI\right) x-\left( \frac{l^{k}+u^k}{2}\right) ^T\left( Q_i+\theta _iI\right) \left( \frac{l^{k}+u^k}{2}\right) \\&\ge   d_i^Tx-\theta _i\sum \limits _{j=1}^n x_j^2+\left( l^{k}+u^k\right) ^T\left( Q_i+\theta _iI\right) x -\left( \frac{l^{k}+u^k}{2}\right) ^T\left( Q_i+\theta _iI\right) \left( \frac{l^{k}+u^k}{2}\right) \\&\ge   d_i^Tx+\theta _i\sum \limits _{j=1}^n\left[ -2l^k_{j}x_{j}+2u^k_{j}l^k_{j}-\left( u^k_{j}\right) ^{2}\right] +\left( l^{k}+u^k\right) ^T\left( Q_i+\theta _iI\right) x\\&\quad -\left( \frac{l^{k}+u^k}{2}\right) ^T\left( Q_i+\theta _iI\right) \left( \frac{l^{k}+u^k}{2}\right) \\&=   \left[ d_i+\left( Q_i+\theta _iI\right) ^{T}\left( l^{k}+u^{k}\right) -2\theta _il^k\right] ^Tx+2\theta _i\left( u^k\right) ^{T}l^k-\theta _i\left( u^k\right) ^{T}\left( u^k\right) \\&\quad -\left( \frac{l^{k}+u^k}{2}\right) ^T\left( Q_i+\theta _iI\right) \left( \frac{l^{k}+u^k}{2}\right) \\&=   \left[ d_i+Q_i^{T}\left( l^{k}+u^{k}\right) +\theta _i\left( u^{k}-l^{k}\right) \right] ^Tx +2\theta _i\left( u^k\right) ^{T}l^k-\theta _i\left( u^k\right) ^{T}\left( u^k\right) \\&\quad -\left( \frac{l^{k}+u^k}{2}\right) ^T\left( Q_i+\theta _iI\right) \left( \frac{l^{k}+u^k}{2}\right) \\&=   F^{L}_i(x). \end{aligned}$$

Therefore, we have

$$\begin{aligned} F^{L}_i(x)\le F_{i}(x),\quad i=0,1,\ldots ,m. \end{aligned}$$

(2) Consider $F_{i}(x)$ and $F^{L}_i(x)$, let $\Delta _{i}=\Vert F_{i}(x)-F^{L}_i(x)\Vert$, we can follow that

$$\begin{aligned} \Delta _{i}& =  \left\| x^TQ_ix+d_i^Tx-\left\{ \left[ d_i+Q_i^{T}\left( l^{k}+u^{k}\right) +\theta _i\left( u^{k}-l^{k}\right) \right] ^Tx+2\theta _i\left( u^k\right) ^{T}l^k\right. \right. \\&\quad\left. \left. -\theta _i\left( u^k\right) ^{T}\left( u^k\right) -\left( \frac{l^{k}+u^k}{2}\right) ^T\left( Q_i+\theta _iI\right) \left( \frac{l^{k}+u^k}{2}\right) \right\} \right\| \\&=   \left\| \left[ \left( x-\frac{l^{k}+u^k}{2}\right) ^T\left( Q_i+\theta _iI\right) \left( x-\frac{l^{k}+u^k}{2}\right) +d_i^Tx-\theta _i\sum \limits _{j=1}^n x_j^2\right. \right. \\&\quad+\left. \left. \left( l^{k}+u^k\right) ^T\left( Q_i+\theta _iI\right) x -\left( \frac{l^{k}+u^k}{2}\right) ^T\left( Q_i+\theta _iI\right) \left( \frac{l^{k}+u^k}{2}\right) \right] \right. \\&\quad\left. -\left\{ \left[ d_i+\left( Q_i+\theta _iI\right) ^{T}\left( l^{k}+u^{k}\right) -2\theta _il^k\right] ^Tx+2\theta _i\left( u^k\right) ^{T}l^k\right. \right. \\&\quad\left. \left. -\theta _i(u^k)^{T}(u^k) -\left( \frac{l^{k}+u^k}{2}\right) ^T\left( Q_i+\theta _iI\right) \left( \frac{l^{k}+u^k}{2}\right) \right\} \right\| \\&=   \left\| \left( x-\frac{l^{k}+u^k}{2}\right) ^T\left( Q_i+\theta _iI\right) \left( x-\frac{l^{k}+u^k}{2}\right) \right. \\&\quad\left. +\theta _i\sum \limits _{j=1}^n\left[ 2l^k_{j}x_{j}-2u^k_{j}l^k_{j}+\left( u^k_{j}\right) ^{2} -x_j^2\right] \right\| \\&=   \left\| \left( x-\frac{l^{k}+u^k}{2}\right) ^T\left( Q_i+\theta _iI\right) \left( x-\frac{l^{k}+u^k}{2}\right) \right. \\&\quad\left. +\theta _i\sum \limits _{j=1}^n\left( u^{k}_{j}-x_{j}\right) \left( x_{j}-l^{k}_{j}+u_j^k-l^{k}_{j}\right) \right\| \\&\le   \left\| \left( x-\frac{l^{k}+u^k}{2}\right) ^T\left( Q_i+\theta _iI\right) \left( x-\frac{l^{k}+u^k}{2}\right) \right\| \\&\quad+\theta _i\left\| \sum \limits _{j=1}^n\left( u^{k}_{j}-x_{j}\right) \left( x_{j}-l^{k}_{j}+u_j^k-l^{k}_{j}\right) \right\| \\&\le   \left\| \left( x-\frac{l^{k}+u^k}{2}\right) ^T\left( Q_i+\theta _iI\right) \left( x-\frac{l^{k}+u^k}{2}\right) \right\| +\theta _i\left\| \sum \limits _{j=1}^n\left( u^{k}_{j}-x_{j}\right) \left( x_{j}-l^{k}_{j}\right) \right\| \\&\quad+\theta _i\left\| \sum \limits _{j=1}^n\left( u^{k}_{j}-x_{j}\right) \left( u_j^k-l^{k}_{j}\right) \right\| \\&\le   \left\| \left( x-\frac{l^{k}+u^k}{2}\right) ^T\left( Q_i+\theta _iI\right) \left( x-\frac{l^{k}+u^k}{2}\right) \right\| +2\theta _i\left\| \left( u^k-l^k\right) ^T\left( u^k-l^{k}\right) \right\| \\&=   \frac{1}{4}\left\| Q_i+\theta _iI\right\| \left\| u^k-l^k\right\| ^2+2\theta _i\left\| u^k-l^k\right\| ^2\\ \end{aligned}$$

Since

$$\begin{aligned} \frac{1}{4}\left\| Q_i+\theta _iI\right\| \left\| u^k-l^k\right\| ^2+2\theta _i\left\| u^k-l^k\right\| ^2\rightarrow 0\ \text{ as }\ \left\| u^k-l^k\right\| \rightarrow 0, \end{aligned}$$

therefore, we get

$$\begin{aligned} \Delta _{i}=\left\| F_{i}(x)-F^{L}_i(x)\right\| \rightarrow 0\ \text{ as }\ \left\| u^{k}-l^{k}\right\| \rightarrow 0. \end{aligned}$$

The conclusion is drawn. $\square$

From the above Theorem 1, we can establish the linear program relaxation problem (LPRP) of the NQPQC in $X^k$ as follows.

$$\begin{aligned} {\mathrm{LPRP}}(X^k):\left\{ \begin{array}{ll} {\mathrm{min}} &{} F^{L}_0(x)\\ {\mathrm{s.t.}}&{} F^{L}_i(x)\le b_i,\quad i=1,\ldots ,m,\\ &{} x\in X^k=\{x:l^{k}\le x\le u^{k}\}. \end{array} \right. \end{aligned}$$

where

$$\begin{aligned}  F^{L}_i(x)&=\left[ d_i+Q_i^{T}\left( l^{k}+u^{k}\right) +\theta _i\left( u^{k}-l^{k}\right) \right] ^Tx+2\theta _i\left( u^k\right) ^{T}l^k -\theta _i\left( u^k\right) ^{T}\left( u^k\right) \\ &\quad -\left( \frac{l^{k}+u^k}{2}\right) ^T\left( Q_i+\theta _iI\right) \left( \frac{l^{k}+u^k}{2}\right) .  \end{aligned}$$

Obviously, by the construction method of linear program relaxation problem (LPRP), we get that the feasible region of the LPRP contain all feasible points of the NQPQC in $X^{k}$, and the optimal value of the LPRP is not more than that of the NQPQC in $X^{k}$.

## Range contraction approach

To accelerate running speed of our algorithm, a range contraction approach is formulated as the following Theorem 2. The proposed range contraction approach aims at contracting the investigated rectangle *X* without pruning any global optimum point of the initial NQPQC.

Without loss of generality, for any $x\in X=(X_{j})_{n\times 1}\subseteq X^{0}$ with $X_{j}=[l_{j},u_{j}]\ (j=1,\ldots ,n)$, we can rewrite the function $F^{L}_i(x)$ as follows:

$$\begin{aligned} F^{L}_i(x)=\sum \limits _{j=1}^n c_{ij}x_j+\delta _{i}, \quad i=0, 1, \ldots , m. \end{aligned}$$

Let $\overline{UB}$ be a known currently upper bound of the global optimum value of the $\hbox {NQPQC}(X^{0}$), and set

$$\begin{aligned} \widehat{LB}_{i}&=  \sum \limits _{j=1}^n\min \{c_{ij}l_{j}, c_{ij}u_{j}\}+\delta _{i}, \quad i=0,1,\ldots ,m,\\ \displaystyle \overline{\overline{X}}_{j}&=   \left\{ \begin{array}{ll} X_{j},&\quad j\in \{1,\ldots ,p-1,p+1,\ldots ,n\},\\ \left(\frac{\overline{UB}-\widehat{LB}_{0}+\min \{c_{0p}l_{p},c_{0p}u_{p}\}}{c_{0p}},u_{p}\right]\bigcap X_{p},&\quad j=p; \end{array} \right. \\ \underline{X}_{j}&=   \left\{ \begin{array}{ll} X_{j},&\quad j\in \{1,\ldots ,p-1,p+1,\ldots ,n\},\\ \, \left[l_{p},\frac{\overline{UB}-\widehat{LB}_{0}+\min \{c_{0p}l_{p},c_{0p}u_{p}\}}{c_{0p}}\right)\bigcap X_{p},&\quad j=p; \end{array} \right. \\ \widetilde{X}_{j}&=   \left\{ \begin{array}{ll} X_{j},&\quad j\in \{1,\ldots ,p-1,p+1,\ldots ,n\},\\ \left(\frac{b_{i}-\widehat{LB}_{i}+\min \{c_{ip}l_{p},c_{ip}u_{p}\}}{c_{ip}},u_{p}\right]\bigcap X_{p},&\quad j=p\ ; \end{array} \right. \\ \widehat{X}_{j}&=  {} \left\{ \begin{array}{ll} X_{j},&\quad j\in \{1,\ldots ,p-1,p+1,\ldots ,n\},\\ \, \left[l_{p},\frac{b_{i}-\widehat{LB}_{i}+\min \{c_{ip}l_{p},c_{ip}u_{p}\}}{c_{ip}}\right)\bigcap X_{p},&\quad j=p\ . \end{array} \right. \end{aligned}$$

### **Theorem 2**

*For any sub-rectangle*$X\subseteq X^{0}$, *the following conclusions hold:*

(i) *If*$\widehat{LB}_{0}>\overline{UB}$*or*$\widehat{LB}_i>b_i$*for some*$i\in \{1,\ldots ,m\}$, *then there contains no global optimal solution of the*$\hbox {NQPQC}(X^{0}$) *overX*.
(ii) *If*$\widehat{LB}_{0}\le \overline{UB}$, *then, for any*$p\in \{1,2,\ldots ,n\}$, *if*$c_{0p}>0$, *then there does not contain global optimal solution of the*$\hbox {NQPQC}(X^{0}$) *over*$\overline{\overline{{X}}}=(\overline{\overline{X}}_{j})_{n\times 1}$; *if*$c_{0p}<0$, *there does not contain global optimal solution of the*$\hbox {NQPQC}(X^{0}$) *over*$\underline{X}=(\underline{X}_{j})_{n\times 1}$.
(iii) *If*$\widehat{LB}_i\le b_i$*for each*$i\in \{1,\ldots ,m\}$, *then, for any*$p\in \{1,2,\ldots ,n\}$, *if*$c_{ip}>0$, *then there does not contain global optimal solution of the*$\hbox {NQPQC}(X^{0}$) *over*$\widetilde{X}=(\widetilde{X}_{j})_{n\times 1}$; *if*$c_{ip}<0$, *there does not contain global optimal solution of the*$\hbox {NQPQC}(X^{0}$) *over*$\widehat{X}=(\widehat{X}_{j})_{n\times 1}$.

### *Proof*

The proof of the Theorem can be similarly given by the Theorem 3 in Jiao et al. (2014), therefore, here is omitted. $\square$

From the above Theorem 2, a new range contraction approach is presented as follows:

### Range contraction approach

Without loss of generality, assume that $X=(X_{j})_{n\times 1}$ with $X_{j}=[l_{j},u_{j}]\ (j=1,\ldots ,n)$ be any sub-rectangle of $X^{0}$, and for each $i=0,1,\ldots ,M,$ compute $\widehat{LB}_{i}$.

If there exist some $i\in \{1,\ldots ,M\},$ such that $\widehat{LB}_{i}>b_{i},$ then delete the whole rectangle *X*;

Otherwise, for each $i\in \{1,\ldots ,M\}$, $p\in \{1,\ldots ,n\},$ compute $\frac{b_{i}-\widehat{LB}_{i}+\min \{c_{ip}l_{p},c_{ip}u_{p}\}}{c_{ip}}.$ If $c_{ip}>0$ and $\frac{b_{i}-\widehat{LB}_{i}+\min \{c_{ip}l_{p},c_{ip}u_{p}\}}{c_{ip}}<u_{p}$, then let $u_{p}=\frac{b_{i}-\widehat{LB}_{i}+\min \{c_{ip}l_{p},c_{ip}u_{p}\}}{c_{ip}}$; else if $c_{0p}<0$ and $\frac{b_{i}-\widehat{LB}_{i}+\min \{c_{ip}l_{p},c_{ip}u_{p}\}}{c_{ip}}>l_{p}$, then let $l_{p}=\frac{b_{i}-\widehat{LB}_{i}+\min \{c_{ip}l_{p},c_{ip}u_{p}\}}{c_{ip}}$.

If $\widehat{LB}_{0}>\overline{UB},$ then delete the whole rectangle *X*;

Otherwise, for each $p\in \{1,\ldots ,n\},$ compute $\frac{\overline{UB}-\widehat{LB}_{0}+\min \{c_{0p}l_{p},c_{0p}u_{p}\}}{c_{0p}}$. If $c_{0p}>0$ and $\frac{\overline{UB}-\widehat{LB}_{0}+\min \{c_{0p}l_{p},c_{0p}u_{p}\}}{c_{0p}}<u_{p}$, then let $u_{p}=\frac{\overline{UB}-\widehat{LB}_{0}+\min \{c_{0p}l_{p},c_{0p}u_{p}\}}{c_{0p}}$; else if $c_{0p}<0$ and $\frac{\overline{UB}-\widehat{LB}_{0}+\min \{c_{0p}l_{p},c_{0p}u_{p}\}}{c_{0p}}>l_{p}$, then let $l_{p}=\frac{\overline{UB}-\widehat{LB}_{0}+\min \{c_{0p}l_{p},c_{0p}u_{p}\}}{c_{0p}}$.

From Theorem 2, by utilizing the above range contraction approach to compress the investigated rectangle region, or delete a part of the investigated rectangle region, in which there does not contain the global optimum solution of the NQPQC. Therefore, we can improve the computational speed or computational efficiency of the algorithm.

## Range division and contraction algorithm

In this section, a new range division and contraction algorithm is presented for globally solving the initial NQPQC. In the algorithm, one of the most important operation is the choice of a suitable range division method. Here, we choose an $w-\hbox {division}$ approach, which is sufficient to ensure the global convergence of the proposed algorithm, this is because that the selected range division method drives all interval to zero for all variables. This range division method is described as follows.

For any investigated rectangle $X^{'}=[\underline{x}^{'},\overline{x}^{'}]\subseteq X^0$. Denote $q\in \arg \max \{\overline{x}_i^{'}-\underline{x}_i^{'}: i=1,2,\ldots ,n\}$, and by dividing the interval $[\underline{x}^{'}_{q},\overline{x}^{'}_{q}]$ into two new subintervals $[\underline{x}_q^{'},\underline{x}_q^{'}+\omega (\overline{x}_q^{'}-\underline{x}_q^{'})]$ and $[\underline{x}_q^{'}+\omega (\overline{x}_q^{'}-\underline{x}_q^{'}), \overline{x}_q^{'}]$, where $\omega \in (0,1).$ At the same time, all other interval remain unchanged. Consequently, the investigated rectangle $X^{'}$ is partitioned into two new sub-rectangles $X^{'}_{1}$ and $X^{'}_{2}$

Without loss of generality, we denote $LB (X^k)$ and $x^k=x(X^k)$ as the optimum value and optimal solution of the linear program relaxation problem over the sub-rectangle $X^k$, respectively. Combining the former linear program relaxation problem, range division method and range contraction approach together, a new global optimization method is constructed for effectively solving the NQPQC, the main steps of the proposed algorithm are described as follows.

### Range division and contraction algorithm

#### *Step 0. (Initializing)*

Set the initial iteration number $k=0$, the initial active node set $\Delta _{0}=\{X^0\}$, the given termination error $\epsilon >0,$ the initial upper bound $UB_{0}=+\infty$ and the initial feasible point set $\Lambda =\emptyset$, respectively.

#### *Step 1. (Judgement)*

Compute the $\hbox {LPRP}(X^{0})$, denote its optimum solution $x^{0}=x(X^{0})$ and optimum value $LB(X^{0})$, respectively. If $x^0$ is a feasible point of the $\hbox {NQPQC}(X^{0}$), let $UB_{0}=F_{0}(x^{0})$ and $\Lambda =\Lambda \cup \{x^{0}\}$. Let $LB_{0}=LB(X^{0})$, if $UB_{0}-LB_{0}\le \epsilon$, then $x^{0}$ is a global optimum solution of the $\hbox {NQPQC}(X^{0}$). Otherwise, proceed to Step 2.

#### *Step 2. (Division)*

 Use the proposed range division method to divide $X^k$ into two new sub-rectangles, and denote the set of new partitioned sub-rectangle as $\overline{X}^k$.

#### *Step 3. (Contraction)*

 For each investigated sub-rectangle $X\in \overline{X}^k$, use the presented range contraction approach to compress its range, and still denote the remaining rectangle part and the remaining partitioning set by *X* and $\overline{X}^k$, respectively.

#### *Step 4. (Bounding)*

 If $\overline{X}^k$ is not empty, for each $X\in \overline{X}^k$, compute the $\hbox {LPRP}(X)$, and denote its optimum value and optimum solution by *LB*(*X*) and *x*(*X*), respectively. If $LB(X)>UB_{k}$, let $\overline{X}^k:=\overline{X}^k{\setminus} X$; else if the midpoint $x^{mid}$ of *X* is a feasible solution of the $\hbox {NQPQC}(X^{0}$), then let $\Lambda :=\Lambda \cup \{x^{mid}\}$, and if *x*(*X*) is a feasible solution of the $\hbox {NQPQC}(X^{0}$), then let $\Lambda :=\Lambda \cup \{x(X)\}$.

If $\Lambda$ is not empty, denote the new upper bound $UB_{k}:=\min _{x\in \Lambda }F_0(x)$, and denote the best feasible point $x^{*}:=\text{ argmin }_{x\in \Lambda }F_0(x)$.

Denote the new remaining partition set and the new lower bound by $\Theta _k:=(\Theta _k{\setminus} X^k)\cup \overline{X}^k$ and $LB_{k}:=\inf _{X\in \Theta _k}LB (X)$, respectively.

#### *Step 5. (Judgement)*

 Denote $\Theta _{k+1}=\Theta _{k}{\setminus} \{X: UB_{k}-LB(X)\le \epsilon ,\ X\in \Theta _k\}$. If $\Theta _{k+1}=\emptyset$, then we have: $UB_{k}$ and $x^{*}$ be the global optimum value and the global optimum solution of the initial NQPQC, resepectively. Otherwise, select a new sub-rectangle $X^{k+1}$ which satisfies $X^{k+1}=\text{ argmin }_{X\in \Theta _{k+1}}LB(X)$, let $k:=k+1$ and return to Step 2.

The above algorithm either terminates after finite iteration or generates an infinite iteration sequence, from the exhaustiveness of the used range division approach, we can follow that all intervals of all variables must shrink to a singleton, i.e., $\Vert u^{k}-l^{k}\Vert \rightarrow 0$. At the same time, the Theorem 1 guarantee that the linear program relaxation problem (LPRP) ($X^{k}$) will infinitely approaches the problem $\hbox {NQPQC}(X^{k}$) as $\Vert u^{k}-l^{k}\Vert \rightarrow 0$.

#### **Theorem 3**

*The above algorithm either finishes finitely at the global optimum point*$x^{*}$*of the initial NQPQC, or produces an infinite iteration sequence*$\{x^{k}\}$, *of which any accumulation point will be the global optimum point of the initial NQPQC*.

#### *Proof*

If the above algorithm finishes finitely at some iteration *k*, obviously, when the algorithm ends, we can get $UB_{k}=v^{*}=LB_{k}$. Therefore, from step of the algorithm, we get that $x^{k}$ is a global optimum point of the initial NQCQP.

If the above algorithm does not finish at finite step, then it must produce an infinite sub-rectangle iteration sequence $\{X^{k}\}$, from the exhaustiveness of division approach, we get that the sub-rectangle sequence $\{X^{k}\}$ must converge to a point. From the characteristics of our algorithm, we can get that $\{UB_{k}\}$ and $\{LB_{k}\}$ are nonincreasing and nondecreasing sequences, respectively. So that $\{UB_{k}-LB_{k}\}$ is a monotonic non-increasing sequence. By the conclusion of Theorem 1, we get that the sequence $\{UB_{k}-LB_{k}\}$ must be convergent to zero. From the structure of our algorithm, for each *k*, we have $LB_{k}\le v^{*} \le UB_{k}$. Therefore, we can get that $\lim _{k\rightarrow \infty }UB_{k}=\lim _{k\rightarrow \infty }LB_{k}=v^{*}.$ From steps of our algorithm, we know that $x^{k}$ is always a feasible point of the initial NQCQP, we can get $UB_{k}=G_{0}(x^{k})$. By the continuity of constrained functions, we get that the limitation point $x^{*}$ of $\{x^{k}\}$ is also a feasible point of the initial NQCQP, and we can get the global optimum value $v^{*}=G_{0}(x^{*})$. Hence, the conclusion is proved. $\square$

## Numerical experiments

In this section, in order to verify the performance of the algorithm proposed in this paper (our algorithm), several numerical examples in recent references are implemented on a Intel(R) Core(TM)2 Duo CPU microcomputer. Although these numerical examples have a relatively small number of variables, they are also quite challenging. The proposed algorithm program is coded in C++ language, all linear program relaxation problems are solved by using simplex approach, and the convergence errors are all set as $\epsilon =10^{-6}$ in our experiments. For all examples, the numerical results of optimal solutions, optimal values and number of iterations obtained by our algorithm and other approaches (Jiao and Chen 2013; Thoai 2000; Shen and Liu 2008; Gao et al. 2005b; Jiao et al. 2014; Shen and Jiao 2006; Wang and Liang 2005; Wang et al. 2004; Shen 2005; Shen and Li 2013) are illustrated in Table 1. The numerical experimental results show that our algorithm can globally solve the NQCQP problem. In Table 1, the notation “Iter.” represents “number of iterations”.

**Table 1:** Numerical comparisons with the known approaches for Examples 1–8

Example	References	Optimum value	Optimum solution	Iter.
1	This paper	−16.000000000	(5.000000000, 1.000000000)	5
	Thoai (2000)	−16.0	(5.0, 1.0)	10
	Jiao and Chen (2013)	−16.000000000	(5.000000000, 1.000000000)	5
2	This paper	6.777777779	(2.000000000, 1.666666667)	14
	Shen and Liu (2008)	6.777782016	(2.000000000, 1.666666667)	40
	Jiao et al. (2014)	6.777778233	(2.0, 1.666666667)	32
	Jiao et al. (2014)	6.777778517	(2.0, 1.666666667)	33
	Wang and Liang (2005)	6.7780	(2.00003, 1.66665)	44
3	This paper	0.500000394	(0.500000000, 0.500000000)	28
	Shen and Liu (2008)	0.500004627	(0.5 0.5)	34
	Wang and Liang (2005)	0.5	(0.5, 0.5)	91
	Wang et al. (2004)	0.5	(0.5, 0.5)	96
	Jiao and Chen (2013)	0.500000442	(0.500000000, 0.500000000)	37
4	This paper	118.383672094	(2.555940349, 3.129963478)	45
	Gao et al. (2005b)	118.383756475	(2.5557793695, 3.13016463929)	210
	Jiao et al. (2014)	118.383672231	(2.555800904, 3.130134268)	61
	Jiao and Chen (2013)	118.383671904	(2.555745855, 3.130201688)	59
5	This paper	−1.162881826	(1.500000000, 1.500000000)	31
	Shen (2005)	−1.16288	(1.5, 1.5)	84
	Jiao and Chen (2013)	−1.162882693	(1.500000000, 1.500000000)	24
6	This paper	1.177125219	(1.177124344, 2.177124344)	12
	Shen and Jiao (2006)	1.177124327	(1.177124327, 2.177124353)	434
	Jiao and Chen (2013)	1.177125051	(1.177124344, 2.177124344)	22
7	This paper	−0.999999102	(2.000000000, 1.000000000)	20
	Shen and Jiao (2006)	−1.0	(2.000000, 1.000000)	24
	Jiao and Chen (2013)	−0.999999410	(2.000000000, 1.000000000)	21
8	This paper	−11.36363579	(1.0, 0.181818133, 0.983332175)	352
	Shen and Li (2013)	−10.35	(0.998712,0.196213,0.979216)	1648
	Jiao and Chen (2013)	−11.363636364	(1.0, 0.181818470, 0.983332113)	420

### *Example 1*

(Jiao and Chen 2013; Thoai 2000).

$$ \left\{ \begin{array}{ll} \min & -x_1^2+x_1x_2+x_2^{2}+x_{1}-2x_{2}\\ {\mathrm {s.t.}}& x_1+x_2\le 6,\\& -2x_{1}^{2}+x_{2}^{2}+2x_{1}+x_{2}\le -4,\\ & 1\le x_1\le 6,\quad 1\le x_2\le 6. \end{array} \right. $$

For Example  1, from numerical results in Table 1, compared with the algorithms in Jiao and Chen (2013), Thoai (2000), use the same logic of the algorithm, our algorithm can obtain the same optimal solution (5.0, 1.0), but our algorithm spends less number of iterations.

### *Example 2*

(Shen and Liu 2008; Jiao et al. 2014; Wang and Liang 2005).

$$\begin{aligned} \left\{ \begin{array}{l} \min \ x_1^2+x_2^2\\ {\mathrm {s.t.}}\ \ \ 0.3x_1x_2\ge 1,\\ \ \ \ \ \ \ \ 2\le x_1\le 5,\ 1\le x_2\le 3. \end{array} \right. \end{aligned}$$

For Example 2, by Table 1, compared with the algorithms in Shen and Liu (2008), Jiao et al. (2014), use the same logic of the algorithm, our algorithm can obtain the same optimal solution with less number of iterations; and compared with the algorithms in Wang and Liang (2005), our algorithm can obtain the better optimal solution with less number of iterations.

### *Example 3*

(Jiao and Chen 2013; Shen and Liu 2008; Wang and Liang 2005; Wang et al. 2004).

$$\left\{ \begin{array}{ll} \min & x_{1}\\ {\mathrm {s.t.}}&4x_{2}-4x_{1}^{2}\le 1,\\ & -x_{1}-x_{2}\le -1,\\ & 0.01\le x_{1}\le 15,\ 0.01\le x_{2}\le 15. \end{array} \right. $$

From the numerical results of Example 3 in Table 1, compared with the algorithms in Jiao and Chen (2013), Shen and Liu (2008), Wang and Liang (2005), Wang et al. (2004), our algorithm can obtain the same optimal solution (0.5, 0.5) as Jiao and Chen (2013), Shen and Liu (2008), Wang and Liang (2005), Wang et al. (2004), but our algorithm spends less number of iterations.

### *Example 4*

(Jiao and Chen 2013; Gao et al. 2005b; Jiao et al. 2014).

$$ \left\{ \begin{array}{ll} \min & 6x^{2}_{1}+4x^{2}_{2}+5x_{1}x_{2}\\ {\mathrm {s.t.}}& -6x_{1}x_{2}\le -48,\\ & 0\le x_{1}\le 10,\ 0\le x_{2}\le 10. \end{array} \right.$$

For Example 4, by the numerical results in Table 1, compared with the algorithms in Jiao and Chen (2013), Gao et al. (2005b), Jiao et al. (2014), our algorithm can obtain better or at least as good as optimal solution and optimal value with less number of iterations.

### *Example 5*

(Jiao and Chen 2013; Shen 2005).

$$ \left\{ \begin{array}{ll} \min &\ -x_1+x_{1}x^{0.5}_{2}-x_{2}\\ {\mathrm {s.t.}}& -6x_1+8x_2\le 3,\\ & 3x_{1}-x_{2}\le 3,\\ & \ 1\le x_{1}\le 1.5,\ 1\le x_{2}\le 1.5. \end{array} \right.$$

For Example 5, by the numerical results of Table 1, compared with the algorithms in Jiao and Chen (2013), Shen (2005), our algorithm can obtain the same optimal solution (1.5, 1.5), but our algorithm spend less number of iterations.

### *Example 6*

(Jiao and Chen 2013; Shen and Jiao 2006).

$$ \left\{ \begin{array}{ll} \min &\ x_{1}\\ {\mathrm {s.t.}} & \frac{1}{4}x_{1}+\frac{1}{2}x_2-\frac{1}{16}x^{2}_{1}-\frac{1}{16}x^{2}_{2}\le 1,\\& \frac{1}{14}x^{2}_{1}+\frac{1}{14}x^{2}_{2}-\frac{3}{7}x_{1}-\frac{3}{7}x_2\le -1,\\ & 1\le x_{1}\le 5.5,\ 1\le x_{2}\le 5.5. \end{array} \right. $$

For Example 6, from the numerical results in Table 1, our algorithm obtains the optimal solution (1.177124344, 2.177124344) after 24 iterations, but the algorithm in Shen and Jiao (2006) obtains the optimal solution (1.177124327, 2.177124353) after 24 iteration, obviously our algorithm has higher computational efficiency than the algorithms in Shen and Jiao (2006); compared with the algorithm in Jiao and Chen (2013), our algorithm can obtain the same optimal solution (1.177124344, 2.177124344), but our algorithm spend less number of iterations.

### *Example 7*

(Jiao and Chen 2013; Shen and Jiao 2006).

$$ \left\{ \begin{array}{ll} \min &\ x_{1}x_{2}-2x_{1}+x_{2}+1\\ {\mathrm {s.t.}}& 8x^{2}_{2}-6x_{1}-16x_{2}\le -11,\\ & -x^{2}_{2}+3x_{1}+2x_{2}\le 7,\\ & 1\le x_1\le 2.5,\\ & 1\le x_2\le 2.225. \end{array} \right. $$

For Example 7, from the numerical results in Table 1, compared with the algorithms in Jiao and Chen (2013), Shen and Jiao (2006), our algorithm can obtain the same optimal solution (2.0, 1.0), but our algorithm spend less number of iterations, obviously our algorithm has higher computational efficiency than the algorithms in Jiao and Chen (2013), Shen and Jiao (2006).

### *Example 8*

(Jiao and Chen 2013; Shen and Li 2013).

$$ \left\{ \begin{array}{ll} \min &\ -4x_{2}+(x_{1}-1)^{2}+x^{2}_{2}-10x^{2}_{3}\\ {\mathrm {s.t.}}& x^{2}_{1}+x^{2}_{2}+x^{2}_{3}\le 2,\\&(x_{1}-2)^{2}+x^{2}_{2}+x^{2}_{3}\le 2,\\ & 2-\sqrt{2}\le x_{1}\le \sqrt{2},\\ & 0\le x_{2}, x_{3}\le \sqrt{2}. \end{array} \right. $$

For Example 8, from the numerical results in Table 1, our algorithm obtains the optimal solution (1.0, 0.181818133, 0.983332175) and the optimal value $-11.363635790$ after 352 iterations, but the algorithm in Jiao and Chen (2013) obtains the optimal solution (1.0, 0.181818470, 0.983332113) and optimal value $-11.363636364$ after 420 iteration, and the algorithm in Shen and Li (2013) obtains the optimal solution (0.998712, 0.196213, 0.979216) and optimal value $-10.35$ after 1648 iteration, obviously our algorithm not only has higher efficiency but also get the better optimal solution and optimal value.

In all, from numerical results for Examples 1–8, compared with Jiao and Chen (2013), Thoai (2000), Shen and Liu (2008), Gao et al. (2005b), Jiao et al. (2014), Shen and Jiao (2006), Wang and Liang (2005), Wang et al. (2004), Shen (2005), Shen and Li (2013), the presented algorithm in this paper can globally solve nonconvex quadratic program with quadratic constraints.

## Conclusion

In this article, a new range division and contraction algorithm is proposed for globally solving nonconvex quadratic program with quadratic constraints (NQPQC). The linear program relaxation problem of the initial NQPQC is constructed by utilizing new linearizing method, which is derived by underestimating all quadratic objective function and constraint functions with linear relaxation functions. By applying the current upper bound and linear program relaxation problem of the NQPQC, a range contraction technique is introduced for improving the computational speed of our algorithm. By successive partition of the initial rectangle region, and by subsequently solving a sequence of linear program relaxation problems, the proposed algorithm converges to the global optimum point of the original NQPQC. Finally, Numerical computational results demonstrate the effectiveness and robustness of our algorithm.
